# Identification and characterisation of the immune response properties of *Lampetra japonica* BLNK

**DOI:** 10.1038/srep25308

**Published:** 2016-04-29

**Authors:** Yinglun Han, Xin Liu, Biyue Shi, Rong Xiao, Meng Gou, Hao Wang, Qingwei Li

**Affiliations:** 1 College of Life Science, Liaoning Normal University, Dalian 116029, China; 2 Lamprey Research Center, Liaoning Normal University, Dalian 116029, China

## Abstract

B cell linker protein (BLNK) is a central linker protein involved in B cell signal transduction in jawed vertebrates. In a previous study, we have reported the identification of a BLNK homolog named Lj-BLNK in lampreys. In this study, a 336 bp cDNA fragment encoding the Lj-BLNK Src homology 2 (SH2) domain was cloned into the vector pET-28a(+) and overexpressed in *Escherichia coli* BL21. The recombinant fragment of Lj-BLNK (rLj-BLNK) was purifiedby His-Bind affinity chromatography, and polyclonal antibodies against rLj-BLNK were raised in male New Zealand rabbits. Fluorescenceactivated cell sorting (FACS) analysisrevealed that Lj-BLNK was expressed in approximately 48% of the lymphocyte-like cells of control lampreys, and a significant increase in Lj-BLNK expression was observed in lampreys stimulated with lipopolysaccharide (LPS). Western blotting analysis showed that variable lymphocyte receptor B (VLRB) and Lj-BLNKwere distributed in the same immune-relevant tissues, and the levels of both were upregulated in supraneural myeloid bodies and lymphocyte-like cells after LPS stimulation. Immunofluorescence demonstrated that Lj-BLNK was localized in VLRB^+^ lymphocyte-like cells. These results indicate that the Lj-BLNK protein identified in lampreys might play an important role in the VLRB-mediated adaptive immune response.

As a lymphocyte subtype of white blood cells, B lymphocytes (B cells) not only are the principal components of the adaptive immune system but also serve various immune functions, such as producing different antibodies and cytokines^1^. The B-cell receptor (BCR) and lipopolysaccharide (LPS) signaling pathways are mainly involved in naïve B-cell activation^2^,^3^. The BCR is a complex that contains membrane immunoglobulin (Ig) molecules and Igα/Igβ (CD79a/CD79b) heterodimers. Once membrane Ig subunits bind antigens, the BCR complex begins to aggregate, and the Igα/Igβ subunits rapidly activate the Src family kinases Lyn, Blk, and Fyn as well as the spleen tyrosine kinase (Syk) and Bruton’s tyrosine kinase (Btk)^1^. The coupling of Syk to several distal substrates requires a linker protein, B cell linker (BLNK)^4^. A typical BLNK sequence encodes an N-terminal leucine zipper motif followed by an “acidic” region, a proline-rich region, and a C-terminal Src homolog 2 (SH2) domain^4^. The leucine zipper motif allows BLNK to localize to the plasma membrane, presumably via coiled-coil interactions with a membrane protein^5^. The “acidic” region of BLNK contains several permanently phosphorylated sites that mediate protein-protein interactions between BLNK and phospholipase Cγ (PLCγ2), Btk, the guanine nucleotide exchange factor Vav (Vav), and the non-catalytic region of tyrosine kinase adaptor protein (Nck)^6^. BLNK recruitment to the plasma membrane also occurs when the SH2 domain binds to a non-immunoreceptor tyrosine-based activation motif (ITAM) phosphotyrosine on Igα^7^,^8^. The activation of BCR signaling leads to BLNK phosphorylation, which in turn recruits PLCγ, BTK, growth factor receptor-bound 2 (Grb2), Vav and Nck to the BCR complex^9^ and initiates multiple signaling cascades involving kinases (p38mitogen-activated protein kinases (p38), c-Jun N-terminal kinases (JNKs) and extracellular-signal-regulated kinases (ERKs)), GTPases, and transcription factors (nuclear factor of activated T-cells (NFAT))^10^,^11^,^12^. These reaction cascades lead to changes in cell metabolism, gene expression, and cytoskeletal organization, which can generate many distinct outcomes, including survival, tolerance (anergy), apoptosis, proliferation, and differentiation into antibody-producing cells or memory B cells^1^.

Lipopolysaccharide (LPS) is a major component of the outer membrane of Gram-negative bacteria and is a prime target for host immune system recognition^13^. The first host protein involved in LPS recognition is LPS-binding protein (LBP)^14^, which has been shown to bind LPS first and then form a ternary complex with CD14. This LPS-LBP-CD14 complex transfers LPS to the LPS receptor complex, which contains Toll-like receptor-4 (TLR4) and myeloid differentiation protein 2 (MD-2)^15^,^16^. The cooperation of LPS-LBP-CD14 with TLR4-MD-2initiates two different signal transduction processes in B cells. One early response is a myeloid differentiation factor 88 (MyD88)/MyD88-like adapter (Mal))-dependent pathway^17^, which activates NF-κB and drives production of tumor necrosis factor alpha (TNF-α), interleukin (IL)-6 or IL-12p40^18^. Another delayed LPS response is a TIR-domain-containing adapter-inducing interferon-β (TRIF)-dependent response, which leads to NF-κB activation and induces interferon regulatory factor 3 and interferon-β expression^19^.

Lampreys and hagfish belong to the class Gnathostomata, which contains extinct and modern jawless vertebrates. As a group of lower vertebrates, Gnathostomata not only share several primitive features, such as the innate immune response system of jawed vertebrates, but also exhibit adaptive immune reactions that involve antigen-specific immunological memory^20^. Although T-cell receptors (TCR) and BCRs are not present in jawless vertebrates, recent findings have revealed that they possess an alternative immune system that specifically recognizes and responds to external pathogens^21^. This alternative immune system uses genomic leucine-rich-repeat (LRR) cassettes for the combinatorial assembly of diverse antigen receptor genes encodinga vast number of variable lymphocyte receptors (VLRs) to resist pathogen invasion^22^,^23^. To date, three types of antigen receptors, VLRA, VLRB and VLRC, have been identified in lampreys^23^, and recent evidence has indicated that VLRA, VLRB and VLRC are expressed in different lymphocyte subsets that resemble the αβT, B and γδT cells of jawed vertebrates, respectively^23^,^24^. After infection by specific pathogens, VLRB lymphocyte-like cells exhibit increased expression of specific VLRB molecules, and the cells begin to secrete VLRB in a manner analogous to Ig secretion by B cells in jawed vertebrates^25^.

Although BLNK has been extensively studied in jawed vertebrates, little is known about BLNK immune function in jawless vertebrates. In a previous study, we have reported the identification of a BLNK homolog named Lj-BLNK in the lamprey *Lampetra japonica*^26^. In this study, the cloning and prokaryotic expression of a truncated Lj-BLNK cDNA fragment encoding the SH2 domain, the purification of recombinant Lj-BLNK fragments (rLj-BLNK), and the generation of polyclonal antibodies (PAbs) against rLj-BLNK are described. Using verified PAbs, the expression pattern of Lj-BLNK in immune-relevant tissues and the co-localization of Lj-BLNK and VLRB were investigated by western blotting, fluorescence activated cell sorting (FACS) analysis and immunofluorescence methods after LPS treatment. Here, we sought to demonstrate the role of Lj-BLNK in the VLRB-mediated adaptive immune response in lampreys.

## Results

### Expression and purification of the lamprey rLj-BLNK protein

**Figure 1:**
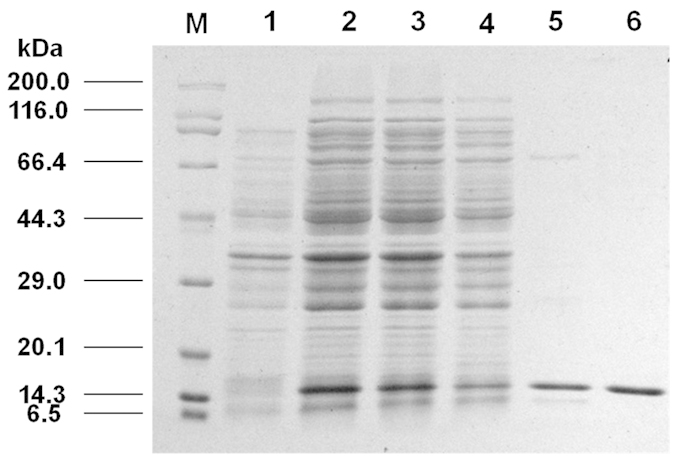
SDS-PAGE analysis and identification of rLj-BLNK protein expressed in *E. coli* BL21. M, Premixed protein marker (Broad); lane 1, Expression ofBL21/pET-28a-BLNKwithout induction; lane 2, Before Lj-BLNK sample purification; lane 3, After Lj-BLNK sample purification; lane 4, Elution of recombinant protein at 50 mM), lane 5, Elution of recombinant protein at 100 mM, lane 6, Elution of recombinant protein at 300 mM.

A 336 bp cDNA fragmentof Lj-BLNK encoding the entire SH2 domain was amplified (Supplementary Fig. S1) and cloned into a pMD19-T vector. A recombinant expression plasmid was reconstructedby inserting the cDNA fragment, which was flanked by *Xho*I and *EcoR*I restriction sites and generated by PCR, into the pET-28a (+) plasmid, as described in the Methods section. After induction with 0.25 mM IPTG, rLj-BLNK was expressed as a soluble His-tagged fusion protein in *E. coli* BL21. The purified rLj-BLNK migrated on a 15% SDS-PAGE gel as a single band with a molecular mass of approximately 14 kDa, which was consistent with the molecular mass predicted from the cDNA sequence (Fig. 1). By MALDI-TOF mass spectrometry analysis, the rLj-BLNK peptide mass fingerprints were identified with significant protein scores (p < 0.05) from Mascot server searches. A peptide with m/z of 1671.5 was determined to be K.GTVYNL.R, which is identical to a fragment of the rLj-BLNK protein sequence (Supplementary Fig. S2). The verified rLj-BLNK was concentrated to 0.5 mg·ml^−1^ for the generation of a polyclonal antibody.

### Titer and specificity analyses of polyclonal antibodies against the truncated rLj-BLNK protein

**Figure 2:**
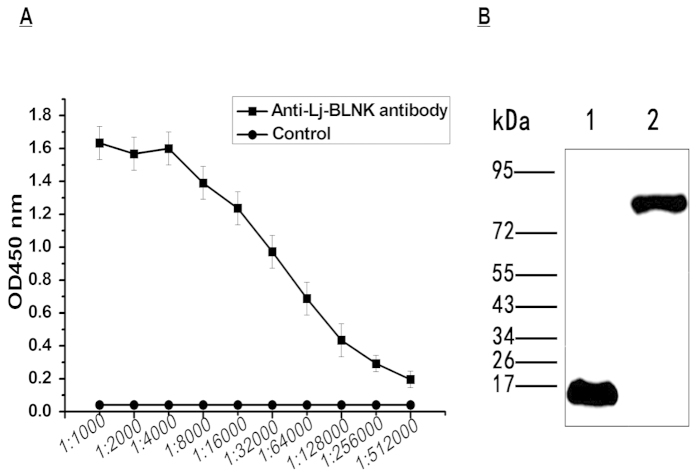
Titer and specificity analysis of a polyclonal antibody against the rLj-BLNK protein. (**A**) Determination of anti-Lj-BLNK polyclonal antibody titer byELISA. The polyclonal antibody against the recombinant protein was diluted from 1000-fold to 512,000-fold. Pre-immunized rabbit IgG was used as a negative control (n = 3). (**B**) Western blotting confirmed the specificity of the anti-Lj-BLNK polyclonal antibody. Lane 1, Purified rLj-BLNK; lane 2, Lymphocyte-like cells (n = 3).

Polyclonal antibodies against the truncated rLj-BLNK protein were purified from rabbit antiserum by CNBr-activated Sepharose 4B affinity chromatography. The ELISA results showed that the titer of rabbit anti-rLj-BLNK PAbs was higher than 1: 512,000 (Fig. 2A). The specificity of the PAbs against Lj-BLNK was determined by western blotting analysis, and the antibody detected both rLj-BLNK and native Lj-BLNK in lymphocyte-like cells (Fig. 2B). Full-length Lj-BLNK was identified as a specific 83 kDa band, which is consistent with the theoretical value (Fig. 2B).

### Expression of Lj-BLNK in VLRB^+^ lymphocyte-like cells

**Figure 3:**
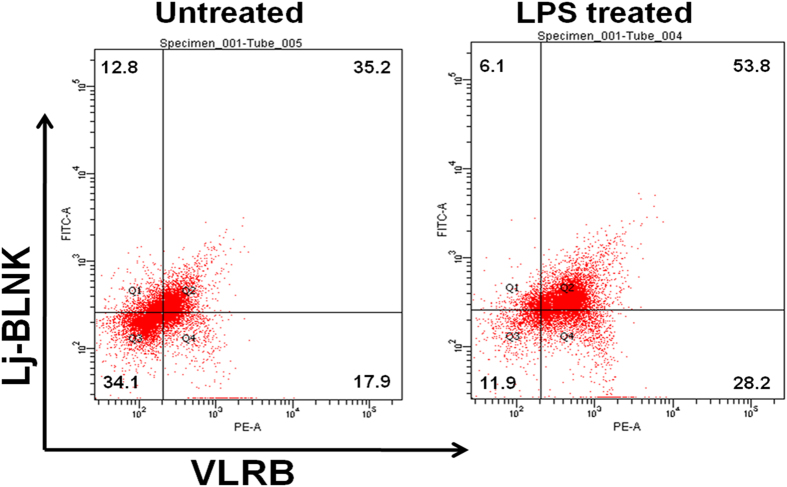
Flow cytometric analyses to detect the expression of Lj-BLNK in lymphocyte-like cells. Lymphocyte-like cells from untreated or LPS-stimulated lampreys were incubated with anti-Lj-BLNK polyclonal antibodies and anti-VLRB monoclonal antibodies and then incubated with FITC-conjugated anti-rabbit IgG secondary antibody and subjected to FACS analysis. Lymphocyte-like cells incubated without antibodies, lymphocyte-like cells incubated with FITC- and PE-conjugated secondary antibodies alone and lymphocyte-like cells bound to polyclonal antibodies from pre-immunized rabbit serum beforeincubating with FITC- and PE-conjugated secondary antibodies served as the controls.

To identify Lj-BLNK expression in VLRB^+^ lymphocyte-like cells, cells from non-treated and LPS-stimulated lampreys were separately incubated with anti-Lj-BLNK and anti-Lj-VLRB Abs and analyzed by FACS. The results showed that Lj-BLNK was expressed in 48% of lymphocyte-like cells (about 74% of VLRB^+^ lymphocyte-like cells) from non-treated lampreys and in 59.9% of lymphocyte-like cells (about 90% of VLRB^+^ lymphocyte-like cells) from LPS-stimulated lampreys (calculated from Fig. 3). The up-regulation of Lj-BLNK expression in VLRB^+^ lymphocyte-like cells after LPS stimulation indicated that Lj-BLNK might be involved in the LPS-induced immune response of lamprey lymphocyte-like cells.

### The expression and localization patterns of Lj-BLNK and VLRB in immune-relevant lamprey tissues

**Figure 4:**
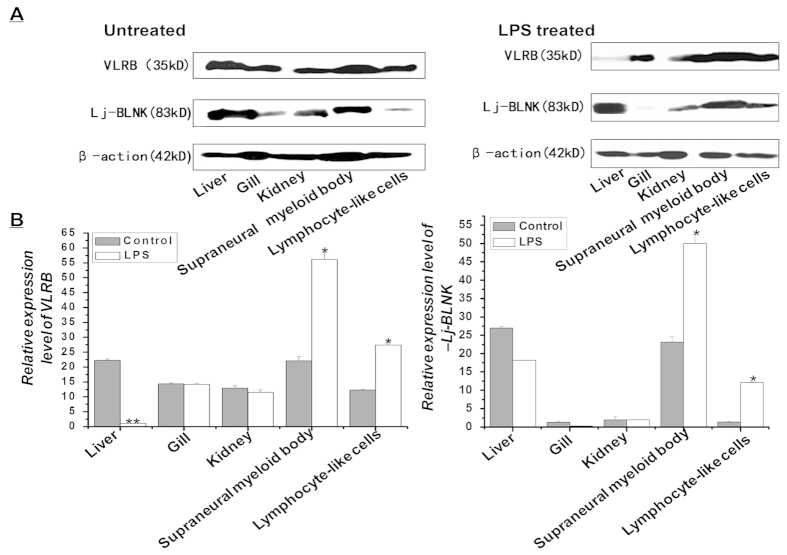
The expression levels of VLRB and Lj-BLNK were determined using western blotting in various immune-relevant tissues. Total protein was extracted from liver, gill, kidney, supraneural myeloid body and lymphocyte-like cells before and after challenge with LPS. Lamprey β-actin served as an internal control, and PBS served as a negative treatment control. Significant differences (p < 0.05) in VLRB and Lj-BLNK expression between the challenged group and the control group are indicated with asterisks (n = 3).

**Figure 5:**
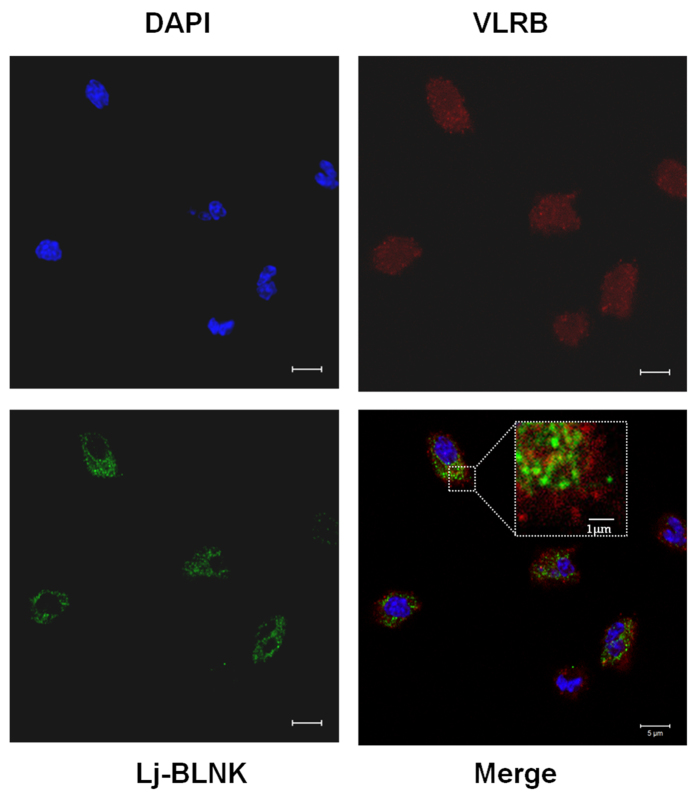
Immunofluorescence detection of VLRB and Lj-BLNK in lymphocyte-like cells. The primary antibodies were rabbit anti-Lj-BLNK and mouse anti-Lj-VLRB (200-fold dilution), and the secondary antibodies were AlexaFluor 488 goat anti-rabbit IgG (400-fold dilution) and AlexaFluor 555 goat anti-mouse IgG (400-fold dilution) conjugated antibodies. The nuclei were stained with DAPI. Scale bar: 5 μm.

The expression patterns of VLRB and Lj-BLNK were examined by using western blotting of total protein extracted from livers, gills, kidneys, supraneural myeloid bodies and lymphocyte-like cells with or without LPS challenge. VLRB and Lj-BLNK were expressed at a much higher level in liver and the supraneural myeloid bodies than in other tissues (Fig. 4). In the LPS-stimulated group, the highest VLRB and Lj-BLNK expression levels were detected in lymphocyte-like cells and supraneural myeloid bodies, in which they were upregulated approximately 2.5-fold compared to corresponding controls (P < 0.05). The significant up-regulation of VLRB and Lj-BLNK in the same immune-relevant tissues such as the supraneural myeloid bodies and lymphocyte-like cells after challenge with LPS indicates that Lj-BLNK might play an important role in the LPS-induced immune response of the VLRB^+^ subset of lymphocyte-like cells. To verify the cellular localization of VLRB and Lj-BLNK, the distribution of VLRB and Lj-BLNK in lamprey lymphocyte-like cells was further evaluated by confocal laser-scanning microscopy. VLRB and Lj-BLNK were distributed on the cell membrane or in the cytoplasm of the same cells (Fig. 5), thus demonstrating that they are co-expressed in the VLRB^+^ subset of lymphocyte-like cells.

## Discussion

In jawed vertebrates, B cell function depends on the ability of BCR to bind an antigen and to effectively induce an efficient biochemical cascade from the cell membrane to the nucleus. Thus, cell morphology is rearranged in the cytosol by cytoskeletal reorganization, and the transcription of new genes is activated in the nucleus to promote cellular differentiation and proliferation. It has been shown that at least three families of protein tyrosine kinases (PTK), including the Src-family of PTKs (including Fyn, Lyn, and Blk), Syk, and Btk, participate in these biochemical events^4^. BLNK was first identified as a new member of the PTK family by Fu *et al.* who have reported that BLNK interacts with PLCγ, Grb2, and Vav after BCR activation^27^. In jawless vertebrates, instead of the Ig fold-based BCR, lymphocyte-like cells express VLRBs containing LRRs that are similar to TLRs in structure but similar to Abs (secreted VLRB) and BCRs (membrane-anchored VLRB) in function^28^. Although the receptors and Abs in jawless vertebrates are well studied, the types of molecules participating in the cascade reaction from signal recognition to Ab production is still unclear. Interestingly, we have recently identified some PTK homologs (Btk, Syk, Fyn, BLNK, and Vav), PLCγ, and Grb2 in the lamprey *L. japonica*^26^,^29^,^30^,^31^. The discovery and validation of these BCR downstream signaling molecules in lampreys indicate that the intracellular signal transduction mechanisms of lamprey VLRB^+^ lymphocyte-like cells and higher vertebrates B cells appear to be conserved, despite differences in the transmembrane signaling pathways (jawless vertebrates lack certain critical immune mediators, such as Igα and Igβ in the BCR complex).

LPS is a major component of the outer membrane of Gram-negative bacteria and has been used in mammals, amphioxus, fish and lamprey for immunological studies^32^,^33^,^34^,^35^. Consistently with these findings, we observed that the expression levels of VLRB and BLNK were upregulated approximately 2.5-fold in lymphocyte-like cells and supraneural myeloid bodies after stimulation with LPS (Fig. 4). It has been well documented that LPS is a potent activator of mature B cells in jawed vertebrates through Toll-like receptor 4 (TLR4) signaling^13^. However, of the 16 lamprey TLR genes and 4 adaptor molecules in the TLR signaling pathwaythat have been identified in the *P. marinus* genome by Kasamatsu *et al.* the critical molecules participating in the B cell response to LPS, including TLR4, TLR9, CD14, MD2, Type I IFN (IFNα/β), TNF-α, IL-6 and IL-12p40, are absent in lampreys^36^. Their results have excluded the possibility that the TLR4-singnaling pathway is responsible for the activation of lymphocyte-like cells by LPS in lampreys, thus raising the possibility that a VLRB-dependent pathway is functionally related to the LPS-induced immune response of lamprey VLRB^+^ lymphocyte-like cells.

The immunization of lamprey with bacteria, viruses, or mammalian cells induces VLRB lymphocytes to undergo lymphoblastoid transformation, proliferation, and differentiation into plasmacytes that secrete antigenreceptors as multivalent VLRB Abs^37^. During the Ab production process, some signaling molecules such as TCR-like, Syk and B cell adaptor protein are upregulated in lamprey VLRB^+^ lymphocyte-likecells^24^. Consistently with these findings, we observed that Lj-BLNK was notably upregulated in VLRB^+^ lymphocyte-like cells after stimulation with LPS (Fig. 3). At the same time, Lj-BLNK was localizedin the cytoplasm of VLRB^+^ lymphocyte-like cells (Fig. 5). The similar expression pattern of VLRB and Lj-BLNK before and after LPS stimulation indicated that Lj-BLNK might play animportant role in the VLRB-mediated signal transduction process of lamprey VLRB^+^ lymphocyte-like cells after LPS stimulation.

Monoclonal lamprey Abs (VLRBs) selectively bind various human glycans, including monosaccharides, disaccharides, trisaccharides, polysaccharides, and glycoproteins^38^,^39^. Although the mechanism of LPS activation in lamprey lymphocyte-like cells remains unknown, it is nonetheless possible that VLRB recognizes LPS with the help of adaptors and, in turn, activates VLRB^+^ lymphocyte-like cells. Thus, further studies are still needed to shed light on the VLRB transmembrane signaling pathway and the recruitment of Lj-BLNK and Lj-BLNK-interacting molecules to understand the mechanism by which Lj-BLNK fulfills its function as an immune molecule in the lamprey adaptive immune system.

## Methods

### Animals and stimulation by LPS

Adult lampreys (*L. japonica*) from the Tongjiang section of the Heilongjiang River (Tongjiang City, Heilongjiang Province, China) were purchased in December. Adult lampreys (200–220 g in weight) were divided into two groups (20 animals per group); one group of animals was immunized with 0.1 mg LPS (*Escherichia coli* 0111:B4, Sigma-Aldrich, St. Louis, MO) in 0.1 ml PBS, andthe other group of animals (control group) was injected with 0.1 ml PBS only. The animals were immunized at 8-day intervals by four intraperitoneal injections and sacrificed with an intraperitoneal injection of ketamine (70 mg kg^−1^) to harvest the tissues for analysis^29^,^40^. Ketamine (Alfasan International B.V, Woerden, The Netherlands) was prepared as 100 mg ml^−1^ in normal saline for use when necessary. This work was approved by the Animal Welfare and Research Ethics Committee of the Institute of Dalian Medical University (Permit Number: SYXK2004–0029), and the methods were performed in accordance with the approved guidelines.

### Expression and purification of rLj-BLNK protein

A 336 bp cDNA fragment encoding the entire Lj-BLNK SH2 domain (GenBank accession number: KF692036) was cloned into a pMD19-T vector (TaKaRa Biotechnology, Dalian, China) as described in the Supplementary Information. This truncated recombinant Lj-BLNK fragment (rLj-BLNK) flanked by *Xho*I and *EcoR*I restriction sites was cloned into the expression vector pET-28a (+) with a His-tag. rLj-BLNK was expressed in *E. coli* BL21with 0.25 mM isopropyl β-D-thiogalactopyranoside (IPTG) (TaKaRa Biotechnology, Dalian, China) induction for 3.5 h. Subsequently, the bacterial cells were harvested by centrifugation at 12000 rpm for 15 min at 4 °C. The cell pellet was suspended in Tris-HClbuffer (50 mM, pH 7.0) containing 20 mM NaCl and 10 mM imidazole, then lysed by sonication. The soluble supernatant was collected and purified with Ni affinity chromatography (GEHealthcare, New York, NY, USA). The concentration of rLj-BLNK was measured using a BCA Protein Assay Kit (Thermo Fisher Scientific, Waltham, MA, USA). Purified rLj-BLNK was analyzed by 15% SDS-PAGE and stored at −20 °C.

### Production of polyclonal antibodies

Purified rLj-BLNK was identified with matrix-assisted laser desorption/ionization time of flight mass spectrometry (MALDI-TOF MS), as described in the Supplementary Information. Polyclonal antibodies against rLj-BLNK were raised in two male New Zealand white rabbits (*Oryctolagus cuniculus*). The rabbits were first immunized with 600 μg of the purified recombinant protein emulsified in Freund’s complete adjuvant (CFA, Sigma-Aldrich, St. Louis, MO, USA) and then boosted three times with 300 μg of protein emulsified in Freund’s incomplete adjuvant (IFA, Sigma-Aldrich, St. Louis, MO, USA) by multipoint intradermal injection once every two weeks. The rabbits were anesthetized with 35 mg kg^−1^ of ketamine three days after a last boost with 600 μg of protein emulsified in IFA, and blood samples were collected from the left common carotid artery. The antiserum samples were collected by centrifugation (12000 × g for 20 min). Polyclonal antibodies against rLj-BLNK were purified with CNBr-activated Sepharose 4B per the manufacturer’s instructions (GE Healthcare, New York, NY, USA). The antibody concentration was adjusted to 1.35 mg ml^−1^, and the antibody was stored at −20 °C in PBS. The titer of the antibodies was determined with an enzyme-linked immunosorbentassay (ELISA). The specificity of the polyclonalantibodies was confirmed by western blotting using rLj-BLNK and lysates from lymphocyte-like cells.

### Fluorescence-activated cell sorting (FACS) analysis

Lymphocyte-like cells isolated from control and LPS-stimulated animals were plated intubes and fixed for 20 mins in a 4% paraformaldehyde solution in PBS at room temperature. Then, the cells were permeabilized with 0.1% Triton X-100 at room temperature for 10 mins. After being washed three times, the cells were blocked with normal donkey serum for 1 h and incubated with rabbit anti-rLj-BLNK polyclonal and mouse anti-VLRB monoclonal antibodies (200-fold) in PBS overnight at 4 °C, then washed three times with PBS. The cells were incubated with FITC-conjugated donkey anti-rabbit IgG (Sigma-Aldrich, St. Louis, MO, USA, 500-fold) for 30 mins at 37 °C in the dark followed by three washes with PBS. The cells were suspended with PBS and analyzed on a FACSAria II flow cytometer (BD Biosciences, San Jose, CA, USA). Cells incubated with FITC-conjugated goat anti-rabbit IgG and PE-conjugated goat anti-mouse IgG were used as isotype controls. Data analysis was performed using FlowJo software (Tree Star).

### Western blotting

Total proteins were separately isolated from the immune-relevanttissues (livers, gills, kidneys, supraneural myeloid bodies and lymphocyte-like cells) of control and LPS-stimulated animalsusing tissue lysis buffer (Beyotime, Shanghai, China). The protein concentrations were determined using a BCA Protein Assay kit (Beyotime, Shanghai, China). The total protein samples from five lamprey tissues were separated by 12% SDS-PAGE and transferred onto polyvinylidene fluoride membranes. The membranes were blocked with 5% BSA (Beyotime, Shanghai, China) and separatelyincubated with rabbit anti-rLj-BLNK (200-fold) or mouse anti-VLRB antibodies (200-fold) overnight at 4 °C; this was followed by incubation with horseradish peroxidase (HRP)-conjugated goat anti-rabbit IgG or HRP-conjugated goat anti-mouse IgG (Sigma-Aldrich, St. Louis, MO, USA, 500-fold)^41^. The membranes were developed using an enhanced chemiluminescent substrate (Beyotime, Shanghai, China). Densitometric analysis was performed using Gel-Pro Analyzer software (Exon-Intron, Inc. Loganville, PA, USA). The optical density data in triplicate from three independent experiments were normalized to lamprey β-actin detected with a rabbit polyclonal antibody against human β-actin (Sigma-Aldrich, St. Louis, MO, USA, 5000-fold) and calibrated to the levels in control samples.

### Immunofluorescence

Lamprey lymphocyte-like cells were fixed and permeabilized as described in the fluorescence activated cell sorting (FACS) analysis section. After being washed three times, cells were blocked with normal goat serum (Beyotime, Shanghai, China) for 1 h and incubated with rabbit anti-Lj-BLNK or mouse anti-VLRB antibodies (200-fold) in PBS overnight at 4 °C, then washed three times with PBS. The cells were incubated with Alexa Fluor 488-conjugated goat anti-rabbit IgG and Alexa Fluor 555-conjugated goat anti-mouse IgG (Sigma-Aldrich, St. Louis, MO, USA, 400-fold). After two additional PBS washes, the lymphocyte-like cells were stained with 4′,6-diamidino-2-phenylindole (DAPI) (Sigma-Aldrich, St. Louis, MO, USA, 200-fold). All of the labeled cells were examined with a confocal laser scanning microscope (LSM710, Zeiss, Oberkochen, Germany) and analyzed using Zeiss ZEN LE software.

### Statistical analysis

All of the detection experiments were performed in triplicate, and the data were expressed as the mean ± SEM. The differences between the two groups were analyzed using Student’s t-test in the SPSS statistical software package. Differences were considered statistically significant at P < 0.05.

## Additional Information

**How to cite this article**: Han, Y. *et al.* Identification and characterisation of the immune response properties of *Lampetra japonica* BLNK. *Sci. Rep.* **6**, 25308; doi: 10.1038/srep25308 (2016).

## Supplementary Material

Supplementary Information
