# Supplementary material for: Identification and characterisation of the immune response properties of Lampetra japonica BLNK
Source: Sci Rep. 2016 Apr 29;6:25308. doi: 10.1038/srep25308 (PMC4850452; doi:10.1038/srep25308)
Supplement: Supplementary Information [file srep25308-s1.pdf]

Title Page

Title: Identification and characterisation of the immune response properties of *Lampetra japonica* BLNK

Author affiliation:

Yinglun Han[1,2#], Xin Liu [1,2#], Biyue Shi [1,2#], Rong Xiao[1,2], Meng Gou[1,2], Hao Wang[1,2], Qingwei Li [1,2*]

[1] College of Life Science, Liaoning Normal University, Dalian 116029, China.

[2]Lamprey Research Center, Liaoning Normal University, Dalian 116029, China

[#] These authors contributed equally to this work and share first authorship.

* Corresponding author: Qingwei Li

Phone and Fax: +86041185822777. E-mail: liqw@263.net

Supplementary Information

SI Methods

Amplification of the coding sequence of the Lj-BLNK SH2 domain. A 336 bp cDNA fragment from Lj-BLNK (GenBank accession number: KF692036) encoding the entire SH2 domain was amplified by using a pair of primers and the template prepared as described below. Total RNA was isolated from lamprey lymphocyte-like cells[1] using RNAiso reagent (TaKaRa Biotechnology, Dalian, China) following the manufacturer's instructions and dissolved in DEPC-treated water. First-strand cDNA was synthesized from 500 ng total RNA with High Fidelity TM Prime Script RT-PCR Kit (TaKaRa Biotechnology, Dalian, China) and used as a template. The PCR primers 5'-CCACCCGAAATAAACAATTGTGAC-3' (forward) and 5'-TCAGATCCTGACCTGATGAATTAG-3' (reverse) were designed based on the *Lj-BLNK* cDNA sequence. The PCR conditions were as follows: 95°C for 30 s; followed by 25 amplification cycles at 95°C for 30 s, 57°C for 60 s, 72°C for 60 s, and a final extension step at 65°C for 5 min. The amplification products were analyzed by electrophoresis on a 2% agarose gel stained with ethidium bromide. The target PCR product band was isolated, purified, and cloned into a pMD19-T vector by using a DNA Ligation kit (TaKaRa Biotechnology, Dalian, China), and the DNA was sequenced (TaKaRa Biotechnology, Dalian, China).

Mass spectrometry of the rLj-BLNK protein. Recombinant Lj-BLNK protein was identified by using a peptide mass fingerprinting technique measured with an Autoflex™ speed MALDI-TOF mass spectrometer (Bruker Daltonics Inc., Billerica, MA, USA). Purified rLj-BLNK was analyzed by 15% SDS-PAGE. The band corresponding to rLj-BLNK was excised from a Coomassie-stained SDS-PAGE gel and digested with sequencing grade modified trypsin (Catalog No: V5111, Promega Corporation, Madison, WI, USA) after the Coomassie stain was removed. The digested peptides in the gel slices were extracted by using the method described by Shevchenko et al[2]. MALDI-TOF mass spectrometry was operated in positive ion mode with the following acquisition cycle: a full scan (m/z 750) recorded in an Orbitrap analyzer at resolution R 60,000, followed by MS/MS of the 20 most intense peptide ions in the LTQ analyzer. All of the MS raw data were searched against all of the lamprey sequences available in the NCBI database by using the MS-Mascot searching algorithm. The search criteria used were as follows: oxidation of Met, carbamidomethylation of Cys, trypsin, 0.5 Da peptide mass to

tolerance, 1 Max missed cleavage sequence coverage > 10%.

SI References

1.Wu, F. et al. A novel BTK-like protein involved in immune response in *Lethenteron japonicum*. *Immunol Lett*. **146**, 57-63 (2012).

2. Shevchenko, A., Tomas, H., Havli, J., Olsen, J.V., Mann, M. In-gel digestion for mass spectrometric characterization of proteins and proteomes. *Nat Protoc*. **1**, 2856–2860 (2006).

Supplementary Fig. S1.

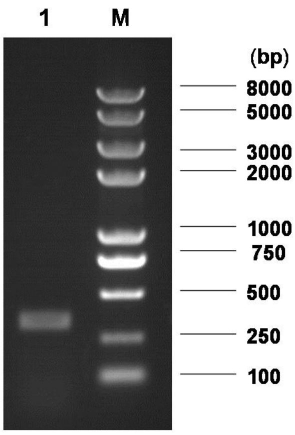

Supplementary Fig. S2.

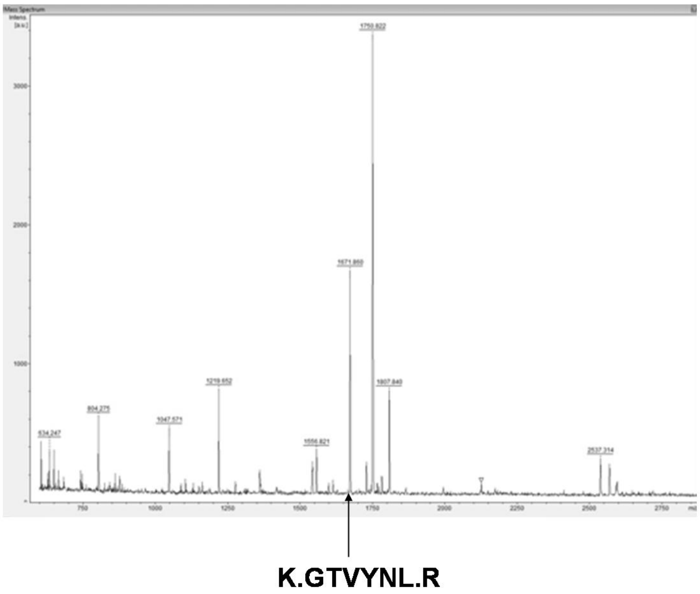

Figure Legends

Supplementary Fig. S1 Amplification of the coding sequence of the SH2 domain of Lj-BLNK. M, DL 8,000 DNA marker; lane 1, A 336-bp cDNA fragment that encodes the SH2 domain of Lj-BLNK.

Supplementary Fig.S2 Peptide mass fingerprinting analysis of recombinant Lj-BLNK protein measured with an Autoflex™ speed MALDI-TOF mass spectrometer. The peptide with m/z 1671.5 corresponds to the tryptic fragment of the complete sequence (K.GTVYNL.R), which is identical to Lj-BLNK (GenBank accessional number: KF692036).
